# Supplementary figures and images for: R2DT: a comprehensive platform for visualizing RNA secondary structure
Source: Nucleic Acids Res. 2025 Feb 8;53(4):gkaf032. doi: 10.1093/nar/gkaf032 (PMC11806352; doi:10.1093/nar/gkaf032)

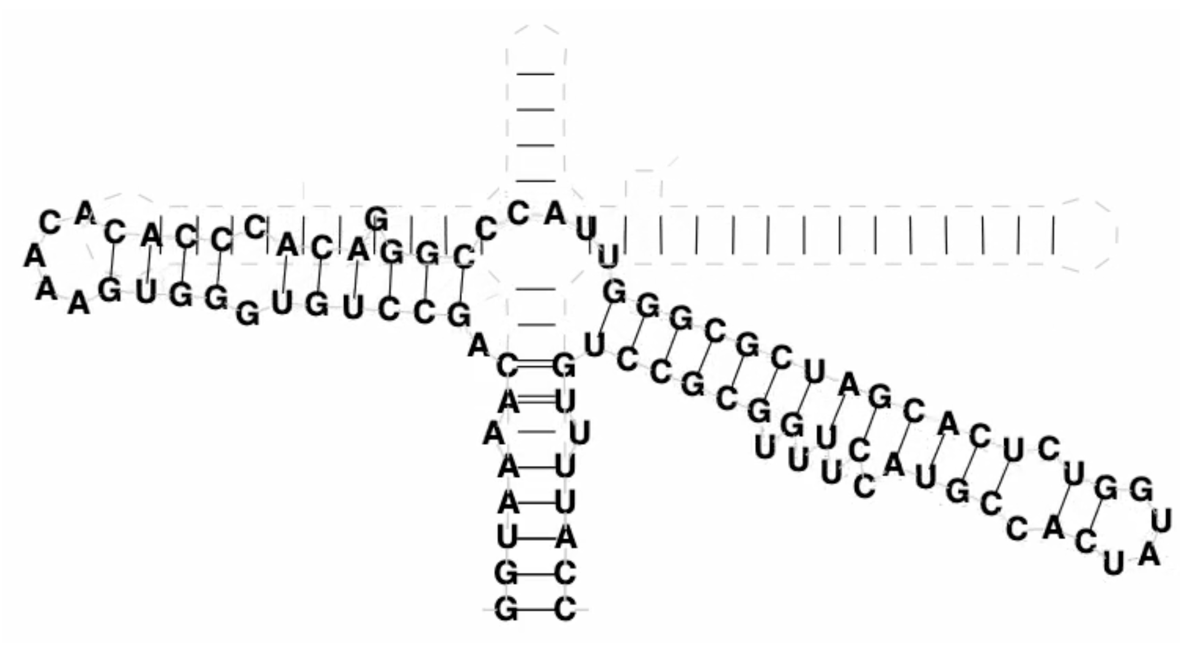

Supplement: gkaf032_Supplemental_Files [file gkaf032_supplemental_files.zip › Video 1.png]
